# DETERMINING APPROPRIATE LOAD: BUILDING STRENGTH AND ENDURANCE AS A REHABILITATION RESEARCHER

**DOI:** 10.1093/geroni/igae098.1524

**Published:** 2024-12-31

**Authors:** Allyn Bove

**Affiliations:** University of Pittsburgh, Pittsburgh, Pennsylvania, United States

## Abstract

Physical therapy educational programs typically teach students to dose patients’ exercises with the goal of increasing strength or increasing endurance – an “either/or” decision rather than a “both/and” decision. However, clinicians working with older adults know that our patients will be more successful when we help them build both strength and endurance. In this presentation, the presenter will discuss why a strategy of building both strength and endurance also applies to the early years of a research career. The presenter will discuss how she built strength while making pivotal career decisions including whether to pursue research or teaching, whether to pursue post-doctoral training, and how much clinical practice is reasonable to build into one’s schedule. Next, the presenter will discuss how she learned (more accurately, ‘is still learning’) to accept being told “no” most of the time, and how the arduous process of pursuing grants and publishing manuscripts builds endurance. Finally, the presenter will discuss lessons learned as an early career rehabilitation researcher. These lessons include: (1) improving balance is difficult, both as a patient in physical therapy and as an early career researcher; (2) it’s okay to be a terrible juggler, as long as one surrounds herself with people who can help her keep all of the balls in the air; (3) how early career researchers may position themselves for careers that are both strong and endurant.

